# A Channelization-Based DOA Estimation Method for Wideband Signals

**DOI:** 10.3390/s16071031

**Published:** 2016-07-04

**Authors:** Rui Guo, Yue Zhang, Qianqiang Lin, Zengping Chen

**Affiliations:** Science and Technology on Automatic Target Recognition Laboratory (ATR), National University of Defense Technology, Changsha 410073, China; zhangyue05@nudt.edu.cn (Y.Z.); even_qqlin@nudt.edu.cn (Q.L.); atrchen@sina.cn (Z.C.)

**Keywords:** digital array radar, direction of arrival (DOA) estimation, wideband signal, digital channelization receiver

## Abstract

In this paper, we propose a novel direction of arrival (DOA) estimation method for wideband signals with sensor arrays. The proposed method splits the wideband array output into multiple frequency sub-channels and estimates the signal parameters using a digital channelization receiver. Based on the output sub-channels, a channelization-based incoherent signal subspace method (Channelization-ISM) and a channelization-based test of orthogonality of projected subspaces method (Channelization-TOPS) are proposed. Channelization-ISM applies narrowband signal subspace methods on each sub-channel independently. Then the arithmetic mean or geometric mean of the estimated DOAs from each sub-channel gives the final result. Channelization-TOPS measures the orthogonality between the signal and the noise subspaces of the output sub-channels to estimate DOAs. The proposed channelization-based method isolates signals in different bandwidths reasonably and improves the output SNR. It outperforms the conventional ISM and TOPS methods on estimation accuracy and dynamic range, especially in real environments. Besides, the parallel processing architecture makes it easy to implement on hardware. A wideband digital array radar (DAR) using direct wideband radio frequency (RF) digitization is presented. Experiments carried out in a microwave anechoic chamber with the wideband DAR are presented to demonstrate the performance. The results verify the effectiveness of the proposed method.

## 1. Introduction

The problem of locating wideband emitters with sensor arrays is of growing interest in many applications such as sonars, radars, advanced satellites and cellular communication systems. Unfortunately, we cannot apply narrowband signal subspace methods such as multiple signal classification (MUSIC) [1] and estimation of signal parameters via rotation invariance techniques ESPRIT [2] on wideband sources because the phase difference between sensor outputs is no longer just dependent on the direction of arrival (DOA) alone, but also depends on the temporal frequency, which has a wide range.

The incoherent signal subspace method (ISM) [3] decomposes wideband signals into many narrowband signals and uses narrowband methods on each decomposed narrowband signals. Then, the results from all the frequency bins are combined to create the final DOA estimation. The ISM method is simple and effective, but it cannot resolve coherent sources and suffers from low signal noise ratio (SNR). The coherent signal-subspace method (CSM) [4] uses a transformation matrix to transform the correlation matrices from many frequency bins into one general correlation matrix at one focusing frequency. Then narrowband signal subspace methods can be applied to estimate the DOA. Although this method has been extended in [5,6] to decrease the resolution threshold and reduce the estimation bias, the method still requires initial DOA estimations and its estimation performance is very sensitive to these initial values. The test of orthogonality of projected subspaces (TOPS) [7] and the test of orthogonality of frequency subspaces (TOFS) [8] are newly introduced methods which estimate DOA by checking the orthogonality between the signal and noise subspaces of different frequency components of the sources. Both TOPS and TOFS are incoherent methods. The performance of TOPS is between that of the coherent and the incoherent methods in the whole SNR range. Recently, TOPS was extended in [9,10] to deal with coherent signals and achieve a higher accuracy. These algorithms perform satisfactorily when the received signals are simple, but this requirement is not guaranteed in real environments. Important parameters including density of signals, dynamic range, frequency range and bandwidth should be taken in consideration. That is why studies in which these algorithms are used in real environments are rare.

The DOA estimation is transformed into a sparse reconstruction problem in recent research [11,12,13,14]. These algorithms have a number of advantages, including increased resolution and improved robustness to noise. However, it is necessary to utilize iteration to achieve a prescribed accuracy in these methods. The intractable computational complexity for sparse recovery makes it difficult to implement on hardware platforms. To the best of our knowledge, there are no wideband DOA estimation methods based on sparse reconstruction that have been validated on hardware platforms. The main purpose of this paper is to present a hardware-efficient wideband DOA estimation method.

The digital channelization receiver shows several desirable characteristics, including high broadband instantaneous frequency coverage, good sensitivity and dynamic range, simultaneous signal detection, arbitration and parameter encoding, and fine frequency measurement [15]. Digital channelization receivers play an important role in radar and electronic warfare applications. The channelization receiver partitions the received signal into a number of sub-channels and arbitrates which sub-channels the signal truly resides in. Afterwards, the parameters of the arbitrated channels including signal center frequency, pulse width and amplitude are then estimated, which is very important for DOA estimation. The channelization receiver architecture was first used to estimate DOA of ultra-wideband signal in [16,17,18]. It splits the array output into multiple frequency channels and down-converts each channel into much lower frequency. Each channel can then be sampled at a lower sampling rate. Each branch of the proposed channelization receiver architecture consists of a mixer, Bessel band-pass filter and low sampling rate analog-to-digital converter (ADC). However, the frequency split and down-convert are implemented before digitization. The performance is highly sensitive to environmental factors, for example, the temperature.

With the development of high-speed ADCs, direct sample to wideband intermediate frequency (IF) signal conversion is possible. Recently, the Science and Technology for Automatic Target Recognition (ATR) Laboratory developed a digital array radar (DAR) test-bed using direct wideband radio frequency (RF) digitization [19,20]. The test-bed works at the IF of 0.6–3.0 GHz. It can achieve an instantaneous bandwidth of 500 MHz. Based on this test-bed, a new DOA estimate flow including IF digitization, digital channelization, channel selection and DOAs estimation is proposed. Firstly, the digital channelization receiver is used to split the wideband array output into multiple narrowband sub-channels. Secondly, the sub-channels within the bandwidth of wideband signals are selected out among the output sub-channels and signal parameters are estimated accurately. Finally, a channelization-based ISM method (Channelization-ISM) and a channelization-based TOPS (Channelization-TOPS) are proposed to estimate the DOA of wideband signals. Channelization-ISM first estimates the DOAs of each sub-channel independently by exploiting the narrowband signal subspace method. Then the estimated DOAs from each sub-channel are averaged to yield the final result. Channelization-TOPS measures the orthogonality between the signal and the noise subspaces of the output sub-channels to subsequently estimate DOAs. Based on the accurate signal parameters estimation, signals with different bandwidths are isolated reasonably. The output SNR is improved by removing the noises from the bandwidth of signals. The proposed channelization method outperforms the conventional ISM method and TOPS method, especially in real environments. Moreover, the output signals in a channelization receiver are in a parallel format, making it easy to implement on hardware.

## 2. The Wideband Signal Model

Assuming a uniform linear array (ULA) of K omnidirectional sensors receives P(P≤K) wideband signals $s_{p}(t)(p=1,2,\cdots P)$ from unknown directions $\left\{\theta_{1},\theta_{2},\cdots ,\theta_{P}\right\}$. The *k*th sensor output $x_{k}(t)$ is:

(1) $$x_{k}\left(t\right)=\sum_{p=1}^{P}s_{p}(t-\tau_{k}(\theta_{p}))+n_{k}(t)\;\begin{array}{c} k=1,2,\cdots K \end{array}$$

where $n_{k}(t)$ is the additive white Gaussian noise of *k*th sensor which is assumed to be statistically independent with signal sources. $\tau_{k}(\theta_{p})$ is the propagation delay of the *k*th sensor:

(2) $$\tau_{k}(\theta_{p})=(k-1)d\sin\theta_{p}/c$$

where c is the speed of the signal propagation, *d* is the spacing between adjacent sensors. The discrete-time Fourier transform (DTFT) of the *k*th sensor output is:

(3) $$X_{k}\left(\omega \right)=\sum_{p=1}^{P}S_{p}(\omega )e^{-j\omega \tau_{k}(\theta_{p})}+N_{k}(\omega )$$

Decomposing the sensor outputs into several narrowband signals using a filter bank or the discrete Fourier transform (DFT), the output can be written in vector format at J frequencies as:

(4) $$X(\omega_{i})=A(\omega_{i})S(\omega_{i})+N(\omega_{i})\;\begin{array}{c} i=0,\cdots ,J-1 \end{array}$$

where:

(5) $$X(\omega_{i})={\left[X_{1}(\omega_{i}),X_{2}(\omega_{i}),\cdots ,X_{K}(\omega_{i})\right]}^{T}$$

(6) $$S(\omega_{i})={\left[S_{1}(\omega_{i}),S_{2}(\omega_{i}),\cdots ,S_{P}(\omega_{i})\right]}^{T}$$

[·]*^T^* denotes the transpose operator. $A(\omega_{i})$ is the *K* × *P* steering matrix. Its columns are the *K* × 1 array manifolds $a(\omega_{i},\theta_{p})$:

(7) $$A(\omega_{i})=\left[a(\omega_{i},\theta_{1}),a(\omega_{i},\theta_{2}),\cdots ,a(\omega_{i},\theta_{P})\right]$$

(8) $$a(\omega_{i},\theta_{p})={\left[e^{-j\omega_{i}\tau_{1}(\theta_{p})},e^{-j\omega_{i}\tau_{2}(\theta_{p})},\cdots ,e^{-j\omega_{i}\tau_{K}(\theta_{p})}\right]}^{T}$$

## 3. The Proposed Method

**Figure 1:**
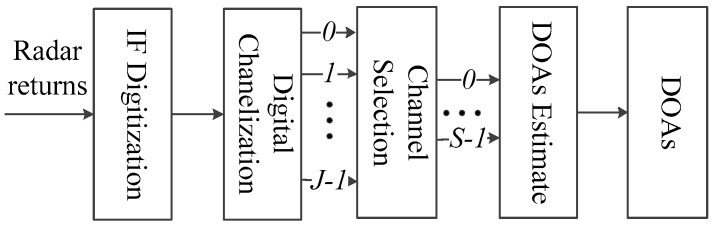
Flow of the proposed method.

Inspired by the digital channelization receiver, we propose a new DOA estimate flow shown in Figure 1, which is composed of IF digitization, digital channelization, channel selection and DOAs estimation. Detailed descriptions of each procedure are presented below. In the DOAs estimation, the channelization-based ISM method and channelization-based TOPS method are proposed.

### 3.1. IF Digitization and Digital Channelization

**Figure 2:**
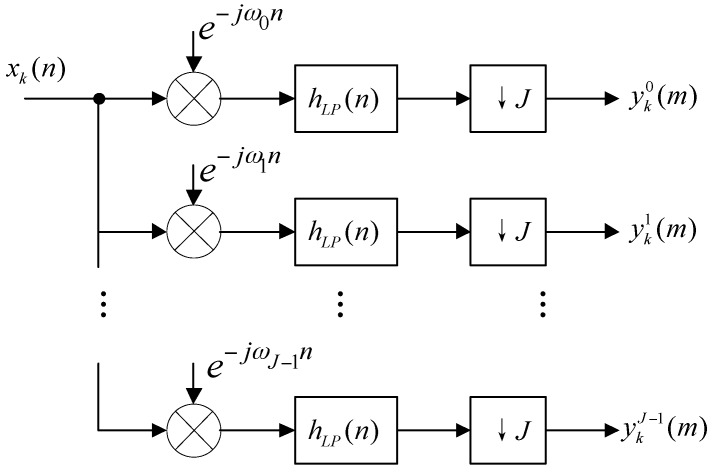
Digital channelization receiver architecture.

The wideband radar returns are directly digitized. A digital quadrature demodulation system [21,22,23] is applied to get the amplitude and phase information of the received signals. A channelization receiver typically employs digital filter banks to extract multiple narrowband sub-channels from the received wideband signals [24,25,26]. The architecture can be illustrated in Figure 2. $x_{k}(n)$ represents the digitization of *k*th sensor output $x_{k}(t)$ with the sampling rate $f_{s}$, where $f_{s}$ is greater than twice of bandwidth of the input wideband signal. ↓ means decimation. The array output is channelized into J frequency channels. The carrier frequency of the *i-*th channel is at:

(9) $$\omega_{i}=\frac{2\pi i}{J}$$

$h_{LP}(n)$ is a low-pass filter.

The output of *i-*th sub-channel can be expressed as:

(10) $$\begin{array}{ll} y_{k}^{i}(m) & =[x_{k}(n)\cdot e^{-j\omega_{i}n}]*h_{LP}(n){|}_{n=mJ}=\sum_{r}x_{k}(n-r)e^{-j\omega_{i}(n-r)}h_{LP}(r){|}_{n=mJ}\\ & =\sum_{r}x_{k}(mJ-r)e^{-j\omega_{i}(mJ-r)}h_{LP}(r) \end{array}$$

The order of prototype filter is D and D=L×J−1, where L is a positive integer. By polyphase decomposition with the prototype filter, Equation (10) can be rewritten as:

(11) $$\begin{array}{ll} y_{k}^{i}(m) & =\sum_{r}x_{k}(mJ-r)e^{-j\omega_{i}(mJ-r)}h_{LP}(r)\\ & =\sum_{p=0}^{J-1}\sum_{l=0}^{L-1}x_{k}(mJ-lJ-p)e^{-j\omega_{i}(mJ-lJ-p)}h_{LP}(lJ+p) \end{array}$$

Define $x_{k}^{p}(m)=x_{k}(mJ-p)$ and $h_{p}(m)=h_{LP}(mJ+p)$. Then:

(12) $$\begin{array}{ll} y_{k}^{i}(m) & =\sum_{p=0}^{J-1}\sum_{l=0}^{L-1}x_{k}(mJ-lJ-p)e^{-j\omega_{i}(mJ-lJ)}h_{LP}(lJ+p)e^{j\omega_{i}p}\\ & =\sum_{p=0}^{J-1}[\sum_{l=0}^{L-1}x_{k}^{p}(m-l)e^{-j\omega_{i}(m-l)J}h_{p}(l)]e^{j\omega_{i}p} \end{array}$$

Substituting Equation (9) into Equation (12), we can rewrite $y_{k}^{i}(m)$ as:

(13) $$\begin{array}{ll} y_{k}^{i}(m) & =\sum_{p=0}^{J-1}[\sum_{l=0}^{L-1}x_{k}^{p}(m-l)e^{-j\omega_{i}(m-l)J}h_{p}(l)]e^{j\omega_{i}p}\\ & =\sum_{p=0}^{J-1}[\sum_{l=0}^{L-1}x_{k}^{p}(m-l)e^{-j\frac{2\pi i}{J}(m-l)J}h_{p}(l)]e^{j\frac{2\pi i}{J}p}\\ & =\sum_{p=0}^{J-1}[\sum_{l=0}^{L-1}x_{k}^{p}(m-l)h_{p}(l)]e^{j\frac{2\pi i}{J}p}\\ & =\sum_{p=0}^{J-1}\left[x_{k}^{p}(m)*h_{p}(m)\right]e^{j\frac{2\pi i}{J}p} \end{array}$$

where ∗ represents convolution operation. Replacing $x_{k}^{p}(m)*h_{p}(m)$ as ${\hat{x}}_{k}^{p}(m)$, then:

(14) $$y_{k}^{i}(m)=J\cdot IDFT({\hat{x}}_{k}^{p}(m))$$

**Figure 3:**
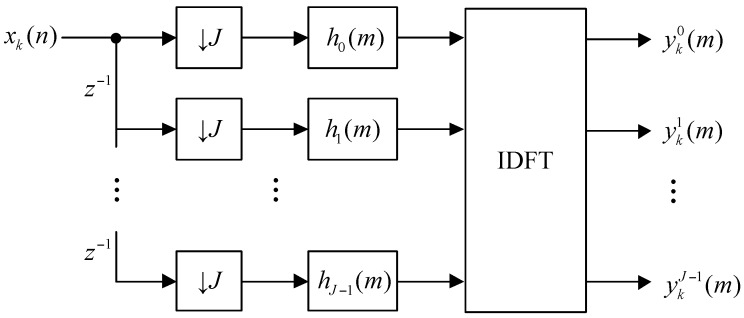
The optimized digital channelization receiver architecture.

Figure 3 shows the optimized digital channelization receiver architecture, which is realized by implementing one low-pass filter and an inverse discrete Fourier transform (IDFT) module. The down sampling by J is moved to the left of the filtering operation. $h_{0}(m)$ to $h_{J-1}(m)$ represent the polyphase components of the low-pass filter $h_{LP}(n)$. This requires fewer components and is a more realistic architecture.

The output of digital channelization receiver $y_{k}^{i}(m)$ can be regarded as a narrowband signal sampled with a sampling rate of $f_{s}/J$. Assume that *N* is the number of snapshots of $x_{k}(n)$ and M that of $y_{k}^{i}(m)$, then we have the conclusion that M=N/J. The bandwidths of the output sub-channels are same. Different output bandwidths are required to deal with more complicated situation in real environments where signals with different unknown bandwidths coexist. The digital channelization receiver architecture can be extended to isolate channels with different bandwidths based on a multistage DFT filter [27,28].

### 3.2. Channelization Selection and Parameter Estimation

The frequency of received signals is not known in a real environment. Sometimes only part of the output sub-channels are within the wideband signal bandwidth. Before estimating the DOA, we need to find the sub-channels in which the wideband signals exist.

The output sub-channels within the bandwidth of wideband signals always result in higher power output. The sub-channel power is a criterion to determine whether there signals exist in the sub-channel. We can compare the power of all the sub-channels to select out the sub-channels within the wideband signal bandwidth.

Assume that $x_{k}(n)$ consists of a wideband signal $s_{k}(n)$ and white Gaussian noise $n_{k}(n)∼N(0,\sigma_{n}^{2})$. $s_{k}(n)$ has a power of $A^{2}$ and the power spreads on S(S<J) sub-channels equally. The input SNR is:

(15) $${\text{SNR}}_{\text{in}}=\frac{A^{2}}{\sigma_{n}^{2}}$$

The output $y_{k}^{i}(m)$ is composed of the response due to the noise $y_{kn}^{i}(m)$ and the signal $y_{ks}^{i}(m)$, respectively. The noise response is a complex filtered baseband noise with an autocorrelation function $\gamma_{y_{kn}}$ [29]:

(16) $$\gamma_{y_{kn}}=E\left[y_{kn}\cdot y_{kn}^{*}\right]=\sigma_{n}^{2}\sum_{n=0}^{N}{\left|h_{k}(m)\right|}^{2}\approx \frac{\sigma_{n}^{2}}{J}$$

Only the S sub-channels within the wideband signal bandwidth are expected in further processing. The SNR of these sub-channels are:

(17) $${\text{SNR}}_{\text{out}}=\frac{A^{2}J}{\sigma_{n}^{2}S}$$

The SNR is improved approximately J/S(J/S>1) times. The signal parameters of each selected sub-channel, including frequency, bandwidth and amplitude must be determined. The DOA of a narrow signal can be estimated with the only sub-channel which the signal resides in. However, wideband signals always span a number of sub-channels. We will introduce how to estimate the DOA of wideband signals in the next section.

### 3.3. Channelization-Based ISM Method

Assume that a total of S sub-channels within the wideband signal bandwidth are selected. Firstly, we use narrowband methods to estimate the DOAs of every selected sub-channel. In practice, the estimated *K* × *K* covariance matrix can be expressed as:

(18) $$\hat{R}(\omega_{i})=\frac{1}{M}\sum_{m=1}^{M}y(\omega_{i},m)y^{H}(\omega_{i},m),\;i=0,1,\cdots ,S-1$$

where ${\left[\cdot \right]}^{H}$ denotes the complex conjugate transpose $y(\omega_{i},m)={\left[y_{1}^{i}(m),\;y_{2}^{i}(m),\;\cdots ,\;y_{K}^{i}(m)\right]}^{T}$. Performing an eigendecomposition on $\hat{R}(\omega_{i})$, the eigenvalues $\lambda_{1},\;\lambda_{2},\;\cdots ,\;\lambda_{K}$ in descending order and their associated eigenvectors $u_{1},u_{2},\cdots ,u_{K}$ can be obtained. Define matrices $F(\omega_{i})=[u_{1},\cdots ,u_{P}]$ and $U(\omega_{i})=[u_{P+1},\cdots ,u_{K}]$, the columns of $F(\omega_{i})$ and $U(\omega_{i})$ span the signal subspace and the noise subspace respectively. DOAs of every sub-channels can be estimated via the spatial spectrum function:

(19) $$P_{SUB}(\omega_{i},\theta )=\frac{1}{{\left\|U^{T}(\omega_{i})a(\omega_{i},\theta )\right\|}^{2}}$$

Then we combine the estimated DOA $\theta_{i}$ for the different sub-channels, and we can find the final DOA estimation. The first estimator exploits the arithmetic mean metric both for the individual sub-channels and the combination over the frequency range, which can be expressed as:

(20) $$P_{Channelized-ISM1}(\omega_{i},\theta )=\frac{1}{\frac{1}{S}\sum_{i=0}^{S-1}\frac{1}{P_{SUB}(\omega_{i},\theta )}}$$

The second estimator uses the arithmetic mean metric for the individual sub-channels and the geometric mean metric for the combination over the frequency range:

(21) $$P_{Channelized-ISSM2}(\omega_{i},\theta )=\frac{1}{{\left(\prod_{i=0}^{S-1}\frac{1}{P_{SUB}(\omega_{i},\theta )}\right)}^{1/S}}$$

The first estimator shows higher accuracy and lower resolution than the second one.

### 3.4. Channelized Based TOPS Method

Following the standard TOPS approach introduced in [7], we first perform an eigendecomposition on $\hat{R}(\omega_{i})$ and obtain the noise subspace $U(\omega_{i})$. Select one output sub-channel as reference to obtain the reference signal subspace $F(\omega_{0})$.

Define a diagonal unitary transformation matrix whose size is k×k and diagonal elements are ${\left[T(\omega_{i},\theta_{i})\right]}_{k,k}$:

(22) $${\left[T(\omega_{i},\theta_{i})\right]}_{k,k}=\exp\{-j\omega_{i}(k-1)d\sin\theta_{i}/c\}$$

${\left[T(\omega_{i},\theta_{i})\right]}_{k,k}$ can transform the array manifold of one frequency into that of another frequency without changing the DOA.

Define a K×P matrix $F_{i}(\phi )$:

(23) $$F_{i}(\phi )=T(\Delta \omega_{i},\phi )F(\omega_{0}),i=1,\;2\ldots ,\;S-1$$

where $\Delta \omega_{i}=\omega_{i}-\omega_{0}$, ϕ is a hypothetic arrival angle of signal:

(24) $$D(\phi )=[F_{1}^{H}U(\omega_{1})|F_{2}^{H}U(\omega_{2})|\ldots F_{S-1}^{H}U(\omega_{S-1})]$$

D(ϕ) is a full rank matrix if ϕ is not equal to the arrival angle of signal otherwise D(ϕ) loses its rank. This theorem holds as long as the source signals are not fully correlated. The estimation of DOA can be obtained through a one-dimensional scan on ϕ, i.e.,

(25) $$\hat{\theta }=\arg\underset{\phi }{\max}\frac{1}{\sigma_{\min}(\phi )}$$

where $\sigma_{\min}(\phi )$ is the smallest singular value of D(ϕ).

### 3.5. Discussion

The channelization receiver shows several appealing characteristics such as wideband frequency coverage, high sensitivity and dynamic range, and accurate measurement of frequency resolution. The digital channelization receiver splits the wideband array output into multiple narrowband sub-channels and meanwhile estimates the parameters of the selected sub-channels. The estimation of the parameters including signal center frequency, pulse width and amplitude is very helpful to DOA estimation. Firstly, signals with different frequencies can be separated into different sub-channels. The DOA estimations of signals in different sub-channels can be obtained accurately without any influence of other signals. Secondly, to estimate the DOA of a signal, the knowledge of the signal parameters such as signal center frequency, pulse width and amplitude is necessary. The channelization receiver offers us a good way to obtain these parameters. Thirdly, the channelization receiver removes noises beyond the bandwidth of signals and selects the sub-channels within the bandwidth of signals with a high accuracy. The output SNR is improved approximately J/S(J/S>1) times. Finally, the channelization receiver can be implemented in a parallel processing architecture, consequently it is able to tackle real-time processing on hardware platforms [29,30]. In virtue of these advantages, the proposed channelization-based method shows a better performance over the conventional ISM and TOPS methods. Compared with the method described in [16], the ADC in the proposed method is closer to the antenna, which can significantly reduce the complexity and hardware cost.

The performances of the proposed method may degrade when the array manifold is affected by mutual coupling, sensor position errors and gain or phase uncertainties. Many calibration algorithms have been developed to compensate these uncalibration impairments [31,32,33] In this paper we assume that the array manifold is deterministic and constant in time. Similar to TOPS, the proposed method works with arbitrary 1-D or 2-D arrays when the array manifolds are linearly independent. Only ULAs are used in the simulation for convenience.

In the DOA estimation procedure, we present the channelization-based ISM method and channelization-based TOPS method. The difference between these two methods is similar to that between the ISM method and TOPS, which is presented in [31]. In this paper, we only focus on the improvement of the proposed channelization-based method instead of the conventional ISM method and TOPS method.

## 4. Simulation

In this section, some computer simulations are conducted to demonstrate the performance of proposed algorithm for DOA estimation of wideband signals. Consider a ULA composed of eight sensors. The maximum number of resolved sources is 7 (K−1). Suppose two wideband signals with the same power located at $\theta_{1}=22{}^{\circ}$ and $\theta_{2}=28{}^{\circ}$ impinging on the ULA. The two sources have the same center frequency $f_{0}=2.65\;\text{GHz}$ and the same bandwidth B=400 MHz. The sampling frequency is chosen to be $f_{s}=1.2\;\text{GHz}$. The sensor noise is complex Gaussian noise.

The average root mean square error (RMSE) of the estimates from 200 Monte Carlo trials is defined as:

(26) $$RMSE=\sqrt{\frac{1}{200}\sum_{t=1}^{200}{\left(\theta_{w}-\theta \right)}^{2}}$$

where $\theta_{w}$ is the DOA estimation value of the incident signal at the *w-*th Monte Carlo trial.

**Figure 4:**
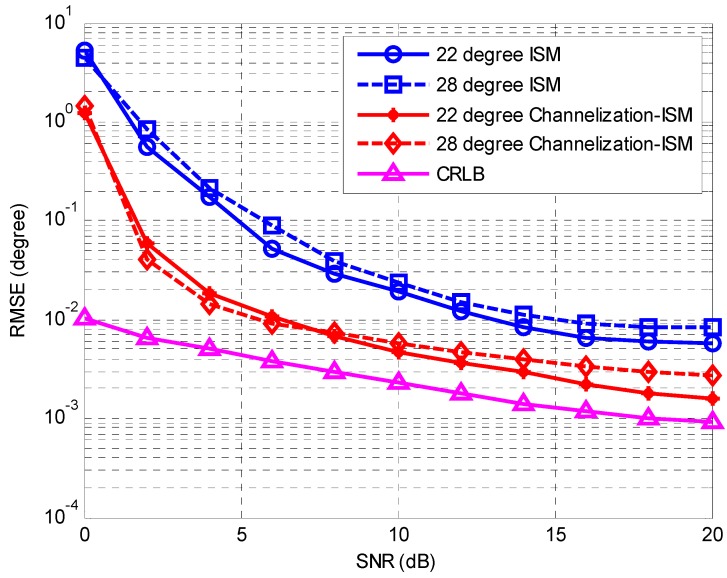
RMSE of the DOA estimates against the SNR.

**Figure 5:**
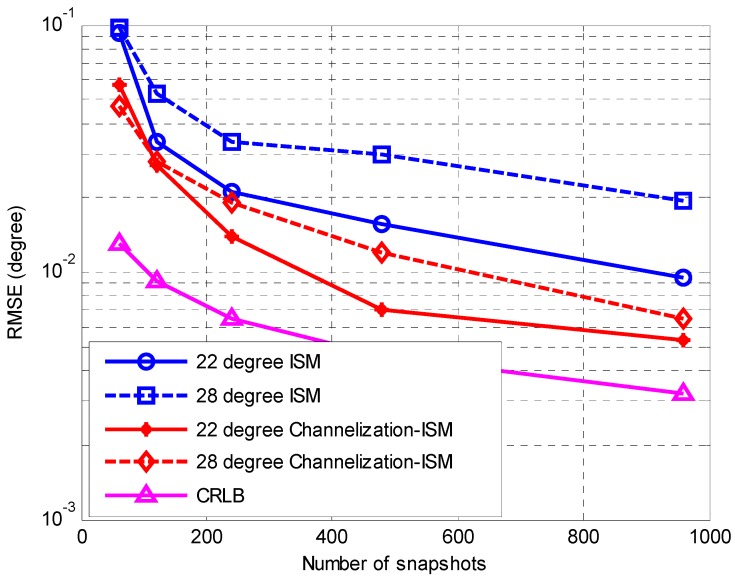
RMSE of the DOA estimates against the number of snapshots.

In the first simulation, the accuracy performances of the ISM method [3] and the proposed Channelization-ISM method are compared. We also present the Cramér-Rao lower bound (CRLB) for the DOA estimations. The first estimator in Equation (17) is applied to yield the final results. The number of sub-channels in the Channelization-ISM method is the same as that of frequency bins in the ISM method, which is 64. The RMSEs against the SNR and the number of snapshots are plotted in Figure 4 and Figure 5, respectively. In Figure 4, the SNR varies from 0 to 20 dB with a step size of 2 dB and the number of snapshots is 500. In Figure 5, the number of snapshots varies from 50 to 1000 and the SNR is fixed at 10 dB. It is clear that with the increase of the SNR and the number of snapshots, the performances of both methods improves. The performance of the proposed method asymptotically approaches CRLB. The proposed Channelization-ISM method shows a better performance than the ISM method at an arbitrary level of SNR and an arbitrary number of snapshots. This is mainly because the channelization receiver has a higher frequency resolution. Signal parameters can be obtained accurately, which is very helpful for DOA estimation. The output SNR is improved by removing by removing the noises beyond the bandwidth of the signals. This result supports the discussions in Section 3.5 and demonstrates the efficiency of our proposed method.

**Figure 6:**
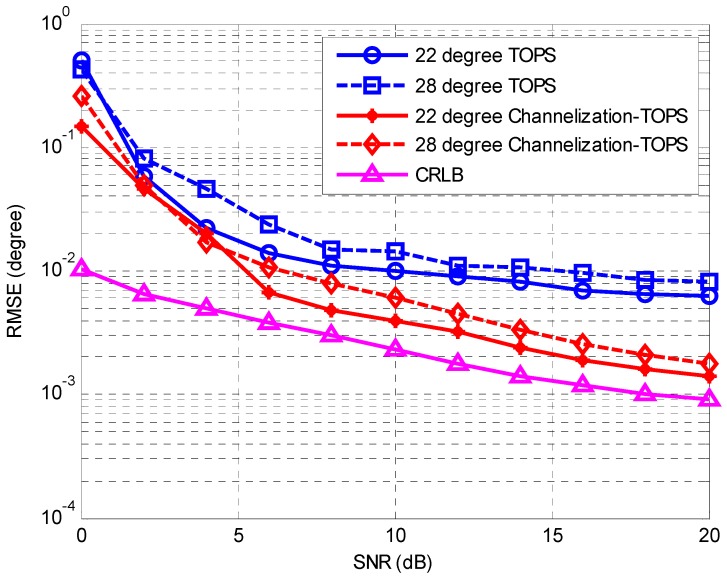
RMSE of the DOA estimates against the SNR.

**Figure 7:**
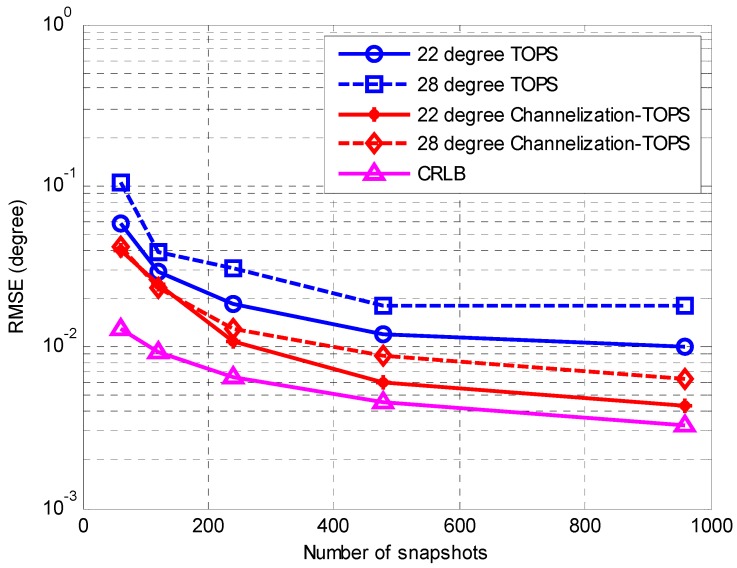
RMSE of the DOA estimates against the number of snapshots.

The second simulation considers the same scenario as the first simulation to compare the performances of the TOPS method [7] and the proposed Channelization-TOPS method. Similar to the result of the first simulation, Figure 6 and Figure 7 show that the proposed Channelization-TOPS method always outperforms the TOPS method at any level of SNR and any number of snapshots. The proposed channelization-based method has a better DOA estimation accuracy than the conventional ISM and TOPS methods.

**Figure 8:**
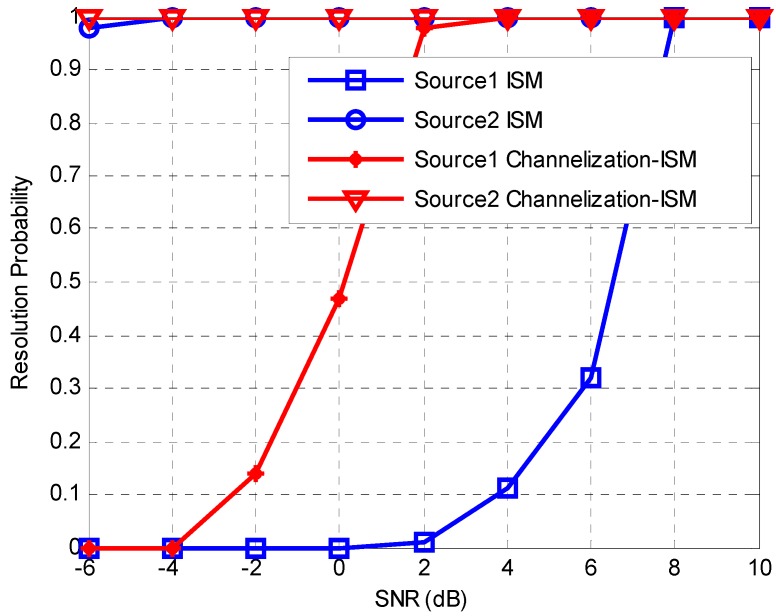
Resolution probability versus SNR with ISM and Channelization-ISM method.

**Figure 9:**
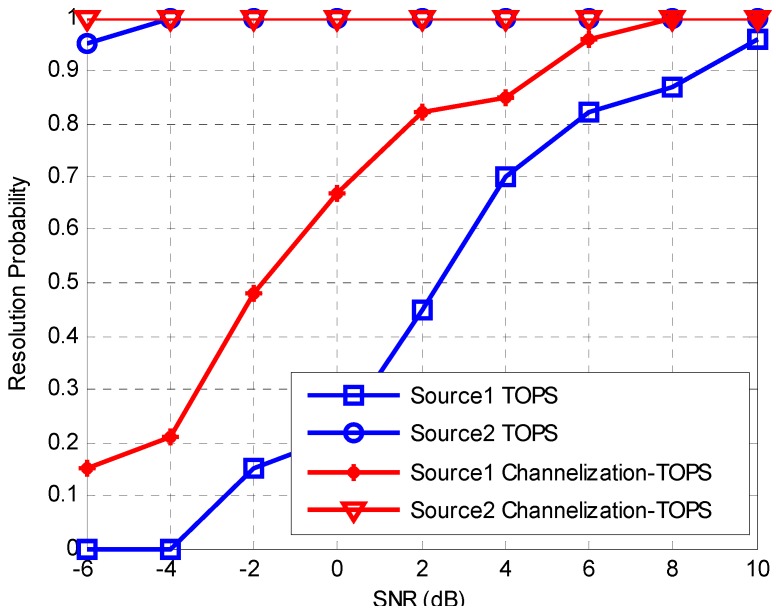
Resolution probability versus SNR with TOPS and Channelization-TOPS method.

To study the detection capability of week signal with the proposed Channelization-ISM method, the resolution probabilities versus SNR for the proposed Channelization-ISM and ISM method are calculated. By definition, a signal is resolved successfully when $\left|{\hat{\theta }}_{k}-\theta_{k}\right|$ is smaller than 0.5, where ${\hat{\theta }}_{k}$ and $\theta_{k}$ represent the estimated and true directions for the *k*th incident signal, respectively. This simulation considers the scenario where there are two sources with one source (weak source) being weaker than the other one by 20 dB. The simulation parameters are the same with those of the second simulation. Figure 8 shows the resolution probability of the two sources versus SNR that corresponds to the strong source with ISM and Channelization-ISM method. Source 1 is the weak source. The resolution probability for the weak source with ISM method is unsatisfactory, as it remains low even though the SNR increases to 6. The proposed Channelization-ISM can obtain similar performance with lower SNR. ISM suffers from bad performance degradation for the weak source while the proposed Channelization-ISM doesn’t. The results illustrate that the proposed channelization-based method performs better on the aspect of dynamic range. Figure 9 which shows the comparison between the TOPS method and Channelization-TOPS also supports the conclusion.

**Figure 10:**
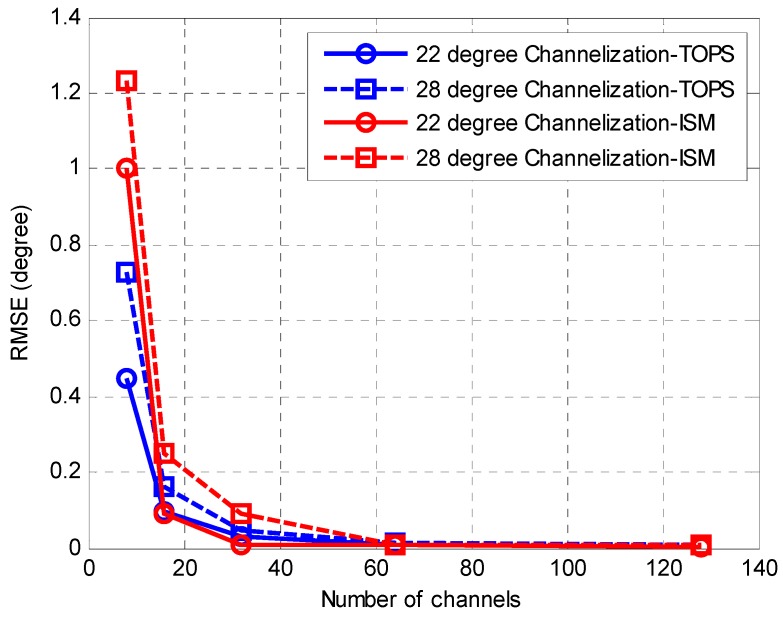
RMSE of the DOA estimates against the number of sub-channels.

In the last simulation, the performances of the proposed Channelization-ISM method and Channelization-TOPS method against the number of sub-channels are studied. The SNR is fixed at 10 dB and the number of snapshots is 256. Figure 10 shows that with the increase of the number of sub-channels, the performances of the proposed methods improves. This is mainly because the sub-channel bandwidth becomes narrower with the increase of the number of sub-channels. Then, the influence over the phase difference between sensor outputs from the temporal frequency of sub-channels becomes weaker. These will lead to a better performance of the DOA estimation. However, the increase of the number of sub-channels may result an increase of the computational complexity. The number of channels used generally is limited when digital channelization is implemented on a hardware platform.

## 5. Experiments

In this section, we present experiments carried out in a microwave anechoic chamber to further demonstrate the performance of the proposed algorithm.

### 5.1. The Architecture of DAR Test-Bed

**Figure 11:**
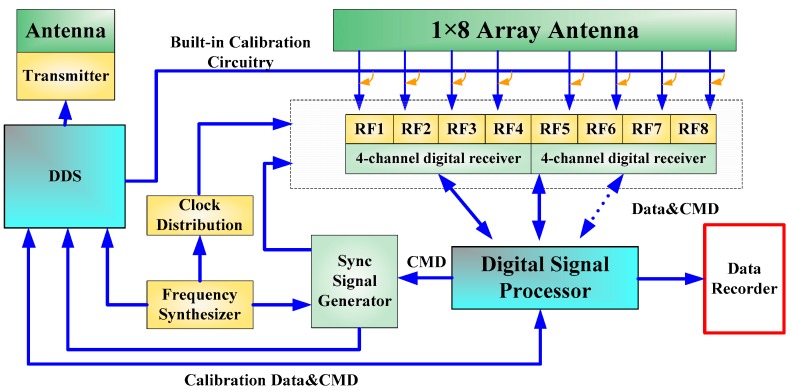
The architecture of the wideband DAR test-bed.

Figure 11 shows the architecture of the wideband DAR test-bed, which is composed of wideband antennas, RF front modules, digital receiver, digital signal processor, built-in calibration circuitry and data recorder.

**Figure 12:**
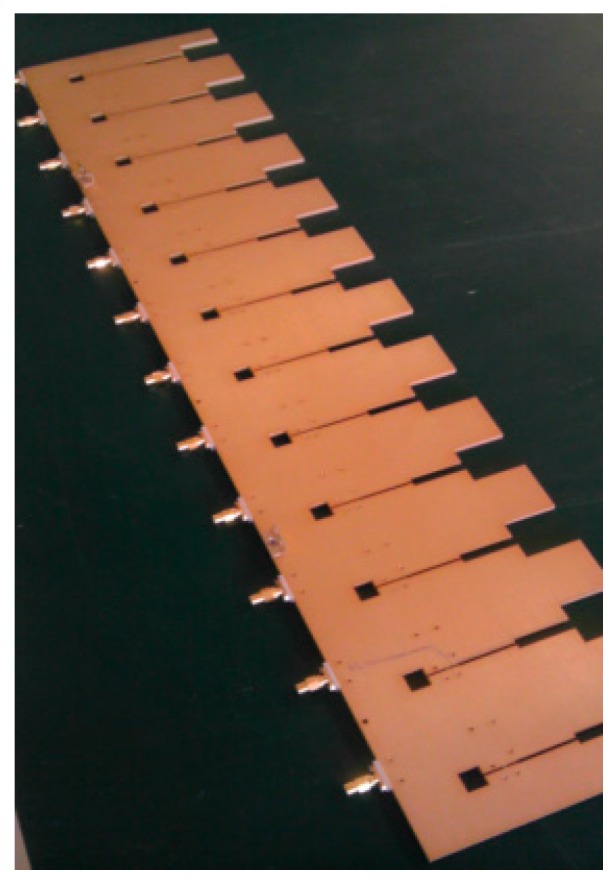
The wideband antenna.

**Figure 13:**
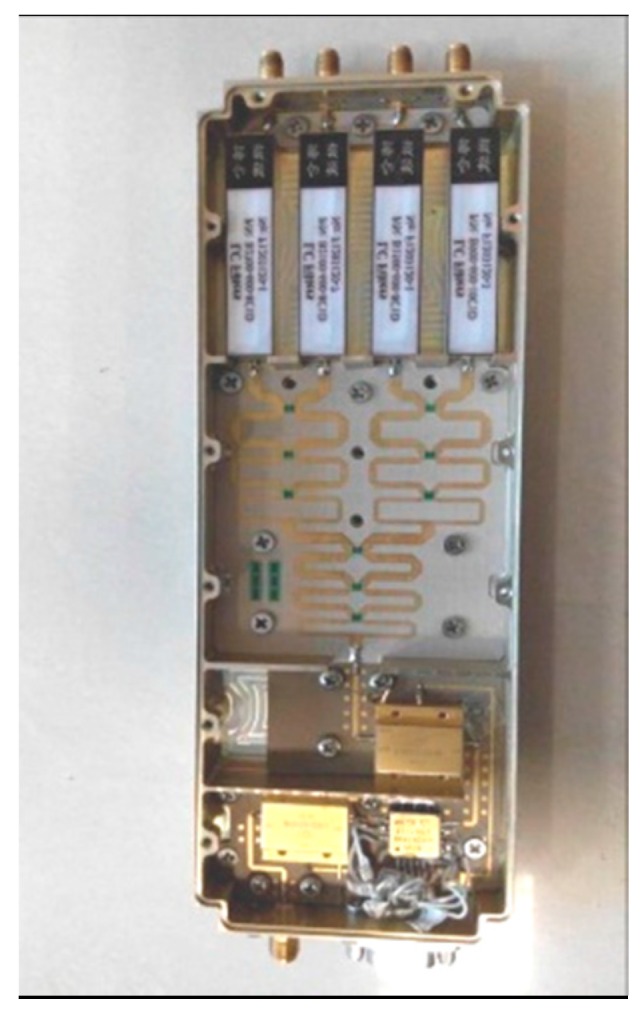
The RF front-end.

**Figure 14:**
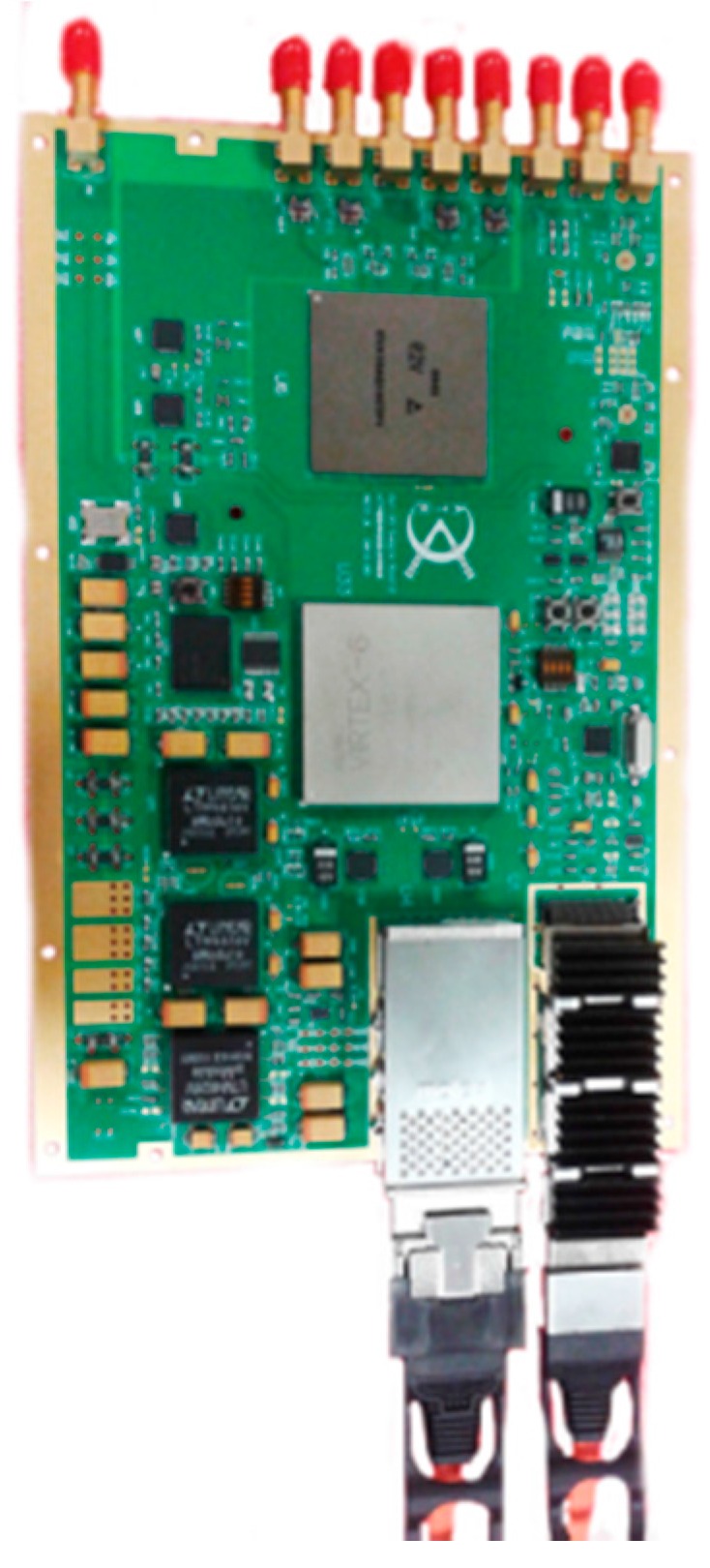
The digital receiver.

**Figure 15:**
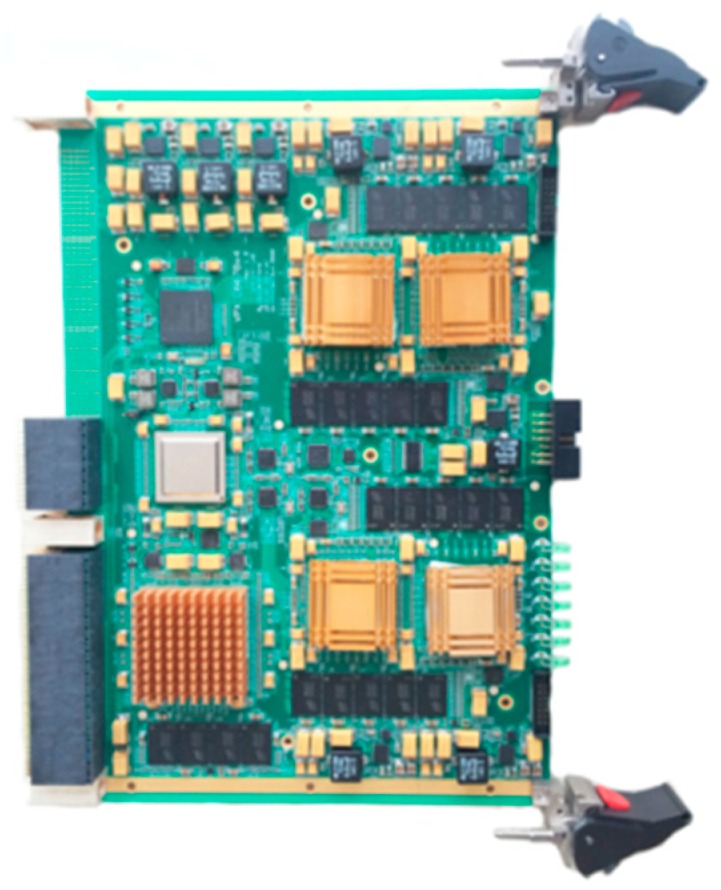
The digital signal platform.

The wideband antenna is realized using eight tapered slot antennas (TSAs) shown in Figure 12. Figure 13 shows the architecture of the RF front-end, which is composed of low noise amplifier (LNA), programmable attenuator, band pass filters, power splitter and RF switch. Signals from the RF front-end are directly digitized by the wideband digital receiver. As shown in Figure 14, the digital receiver consists of ADC, large-scale field programmable gate array (FPGA) and multichannel optical modules. It is capable of directly sampling four input signals up to 3 GHz at the sampling rate of 1.2 GHz utilizing band-pass sampling theories. The digital signal processor shown in Figure 15 is based on the latest generation of high-performance multicore fixed and floating-point DSP TMS320C6678 and Xilinx Virtex-6 family’s high-density, FPGA, which is applicable for high-speed data communication, processing. The data recorder is designed to store kinds of data for system test and post-processing.

### 5.2. Experiment Carried Out in Microwave Anechoic Chamber

**Figure 16:**
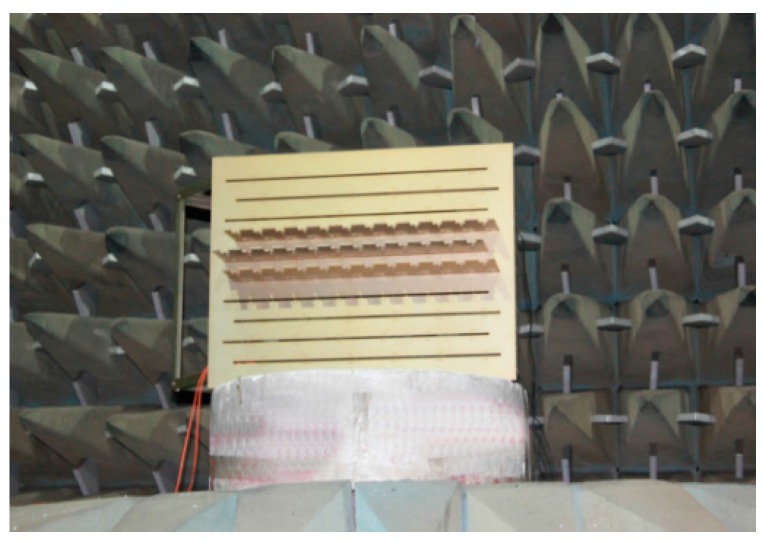
Experiment in a microwave anechoic chamber.

**Figure 17:**
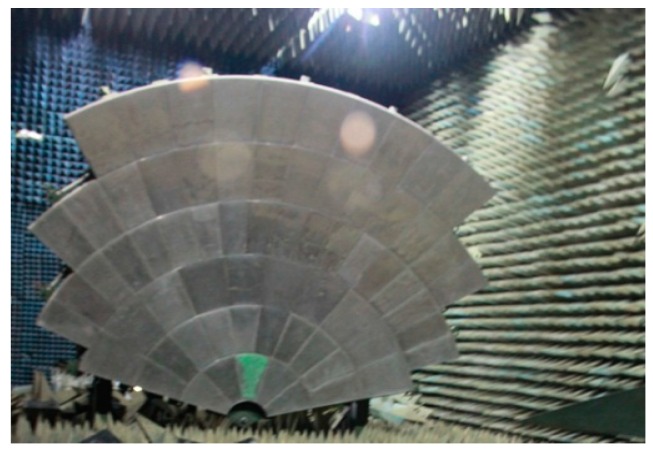
The reflector.

The experiment is carried out in a microwave anechoic chamber. As shown in Figure 16, the antenna array is placed on a rotation platform. Wideband signals impinging on the array are transformed into parallel waves through a reflector which is shown in Figure 17. There center frequency *f*_0_ = 2.7 GHz and bandwidth *B* = 500 MHz. The gain-phase and mutual coupling errors are calibrated by the method given in [34]. The wideband signals are directly sampled by the 10 bits ADCs with a sampling rate of 1.2 Gsps utilizing the band-pass sampling theory. The complex signal data speed is 600 MHz after digital quadrature demodulation. Then we split the signal into 32 sub-channels with the FPGA on digital signal platform. The output data speed of each sub-channel is 18.75 MHz, which is lower than the highest frequency of the Virtex-6 FPGA. Besides, the hard resources consumption is calculated as shown in Table 1. The results illustrate that the hardware platform adequately meets the resources constraint.

**Figure 18:**
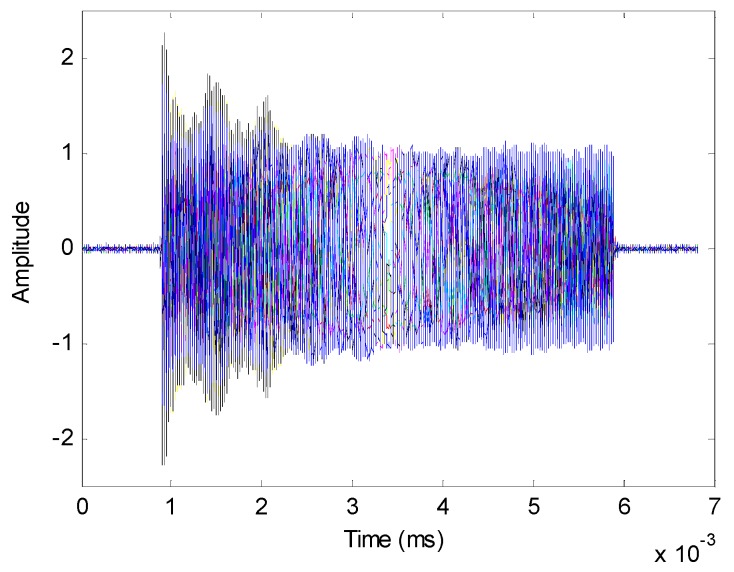
Received signals expressed in time domain.

**Figure 19:**
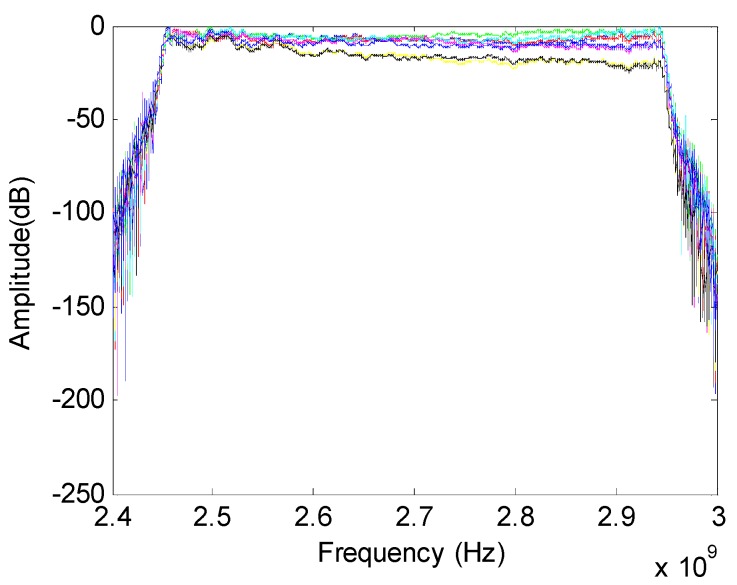
Received signals expressed in frequency domain.

**Figure 20:**
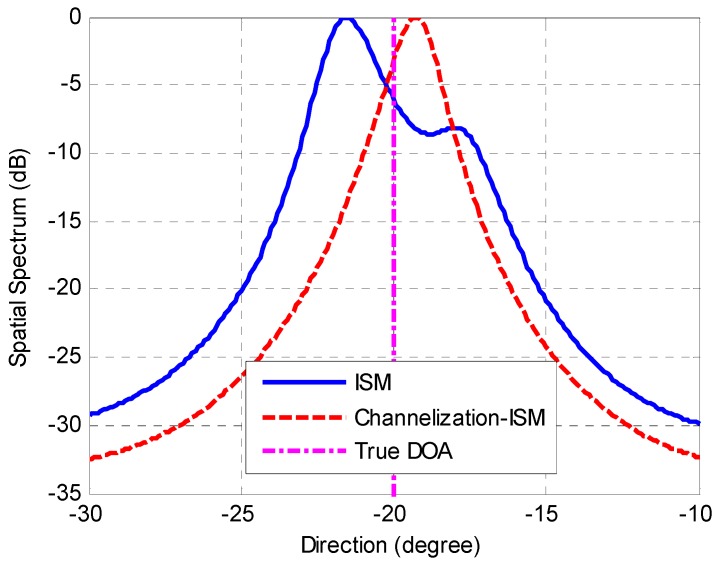
DOA estimations with ISM method and Channelization-ISM method.

**Figure 21:**
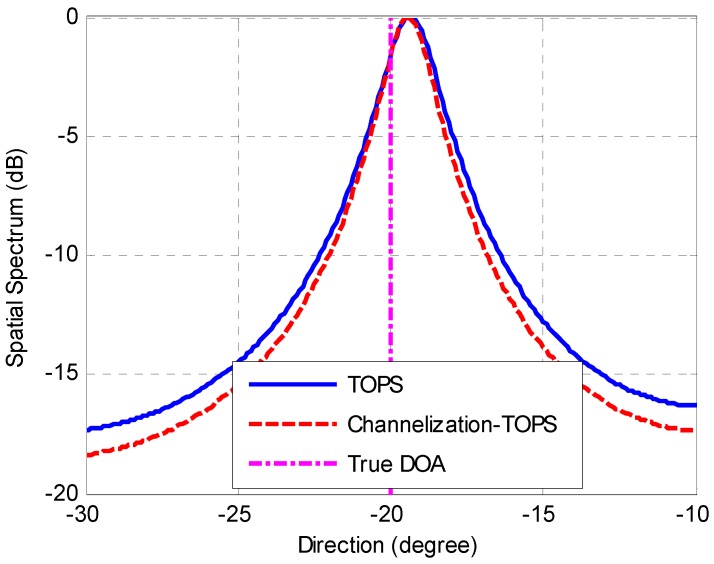
DOA estimations with TOPS method and Channelization-TOPS method.

Figure 18 shows the amplitude of received signals in time domain. Eight curves with different colors represent the different sensor outputs of the ULA. Figure 19 depicts the received signals in the frequency domain. The spatial spectrum estimations of the received signals with different methods are plotted in Figure 20 and Figure 21. The true DOA could be measured according to the records of the rotation platform with a precision of 0.05°. It is clear that the DOA estimations of channelization-based methods are more approximate to the true DOA. The performances of the proposed channelization-based methods are better than those of the conventional ISM and TOPS methods. Rotating the array from −50° to 50° at the step of 1°, 101 groups of original data are obtained. DOA estimations with different incident angles are calculated using different methods. Table 2 presents the RMSEs of 101 estimates with different methods. The proposed channelization methods outperform the conventional ISM and TOPS methods. The experimental results are in good agreement with the simulation results.

## 6. Conclusions

In this paper, we propose a hardware-efficient, channelization-based DOA estimation method for wideband signals. It splits the wideband array output into multiple frequency sub-channels and estimate signal parameters of each sub-channel. The sub-channels within the wideband signal bandwidth are selected on the basis of the power of every sub-channel. The channelization-ISM method applies narrow techniques to the selected channels and gets the final DOA estimate by averaging the results from all the selected channels. The Channelization-TOPS method measures the orthogonality between the signal and the noise subspaces of the output sub-channels to estimate DOAs. The proposed channelization methods outperform the conventional ISM method and TOPS method, which results from the proposed channelization method being able to estimate the signal parameters more accurately and improve the output SNR more. Besides, the parallel processing architecture makes it easy to implement on hardware. Simulations and experiments in a microwave anechoic chamber are carried out as well. The results illustrate that the proposed method can achieve better estimation accuracy and dynamic range performance than the conventional ISM method and TOPS method. 

## Figures and Tables

**Table 1:** Hardware resources consumption.

Hardware Resources	Total	Consumption	Percentage
occupied Slices	49,200	2456	4.99%
Slice Registers	393,600	10,523	2.67%
Slice LUTs	196,800	6618	3.36%
DSP48E1s	1344	477	35.49%
RAMB36E1/FIFO36E1s	704	56	7.95%

**Table 2:** Mean deviation of different methods.

Method	RMSE
TOPS Method (degree)	0.6792
Channelization-TOPS Method (degree)	0.6121
ISM Method (degree)	3.2509
Channelization-ISM Method (degree)	0.9103
